# Chromosome-level reference genome of *Tylorrhynchus heterochaetus* (Annelida, Nereididae)

**DOI:** 10.3389/fgene.2026.1753621

**Published:** 2026-01-28

**Authors:** Wei Yang, Xuemin Zhang, Bin Fan, Yuanyuan Si, Ruiwen Xu, Shengkang Li, Zining Meng, Xinghan Chen

**Affiliations:** 1 Food and Environmental Engineering Department, Yangjiang Polytechnic, Yangjiang, China; 2 Guangdong Provincial Key Laboratory of Marine Biotechnology, Shantou University, Shantou, China; 3 State Key Laboratory of Biocontrol, Institute of Aquatic Economic Animals, and the Guangdong Province Key Laboratory for Aquatic Economic Animals, Life Science School, Sun Yat-Sen University, Guangzhou, China

**Keywords:** chromosomal assembly, gene family comparison, genome annotation, phylogenetic analysis, *Tylorrhynchus heterochaetus*

## Abstract

The *Tylorrhynchus heterochaetus*, a polychaete benthic invertebrate belonging to the Nereididae family, has emerged as a promising aquaculture species. It is highly regarded for its nutritional profile, with protein accounting for up to 60% of its dry weight, as well as its balanced amino acid composition. This has earned it the nickname “aquatic cordyceps”. However, wild populations of this species have declined significantly due to environmental shifts and human activities, with local extinctions reported in certain regions. A critical barrier to advancing its population genetics and conservation biology has been the absence of a chromosomal-level reference genome for *T. heterochaetus*. To address this gap, we present the first chromosome-level genome assembly of *T. heterochaetus*, generated using PacBio HiFi sequencing data and Hi-C technology. The final assembly spans 782.25 Mb with a scaffold N50 of 75.39 Mb, successfully anchored to 11 pseudo-chromosomes. Repetitive sequences account for 428.09 Mb (54.73%) of the genome, and 20,145 protein-coding genes were annotated. This study provides foundational insights into the genetics, genomics, and evolutionary history of *T. heterochaetus*, laying a critical groundwork for future research and enabling the development of targeted genetic conservation strategies.

## 1 Introduction

*Tylorrhynchus heterochaetus*, commonly known as “Hechong” in Chinese, is a polychaete, benthic invertebrate belonging to the Nereididae family (order: Polychaeta; phylum: Annelida). It is widely distributed across the brackish coastal waters of China, Japan and Southeast Asia (Tuan, 2018; Yang et al., 2020). In the estuarine regions of south-eastern China, this species primarily colonises paddy ecosystems, where it feeds on crop roots, forming a natural symbiotic association with rice (Su et al., 2016). The “rice + *T. heterochaetus*” integrated ecological farming model not only significantly increases the economic returns of rice cultivation, but also promotes the reuse of abandoned farmland and helps to ensure food security. Collectively, these attributes make it a promising candidate for emerging aquaculture development.

In addition, *T. heterochaetus* has remarkable nutritional value. Protein accounts for up to 60% of its dry weight and it has a well-balanced amino acid profile (Zhang et al., 2022). This has earned it the reputation of being the “aquatic cordyceps” (Glasby and Timm, 2008). Studies have shown that it contains 10–20 species of fatty acids, B vitamins and trace elements (including calcium, iron and selenium), which together regulate dyslipidaemia and prevent atherosclerosis (Suzuki and Gotoh, 1986; Osanai, 1978). Furthermore, it contains high levels of fibrinolytic enzymes, fibrinogen activators and collagenase, which support the prevention and treatment of cerebral thrombosis and myocardial infarction (Wu et al., 2006). However, *T. heterochaetus* occupies a narrow ecological niche and is acutely sensitive to abrupt environmental shifts. In recent years, rising commercial demand coupled with increasing pressures from water pollution and habitat fragmentation has led to a continuous decline in wild populations and even local extinctions in some regions (Tuan, 2018; Chen et al., 2020).

Currently, research on *T. heterochaetus* has primarily focused on its morphological characteristics, life history, reproductive biology, culture techniques, and nutritional composition (Wu et al., 2006; Sato and Osanai, 1990; Ma et al., 2014). However, systematic genetic investigations remain scarce. To date, only Chen et al. (Chen et al., 2020) have sequenced its complete mitochondrial genome and analyzed the genetic structure of seven geographic populations using mitochondrial COI sequences. Meanwhile, Yang et al. (Yang et al., 2023a; Yang et al., 2023b) leveraged genome survey data and transcriptome information to preliminarily characterize microsatellite features and develop polymorphic molecular markers. Despite these contributions, a complete and well-assembled nuclear genome is still unavailable. This paucity of genomic data significantly impedes in-depth studies of its genetic regulatory networks, adaptive evolution mechanisms, and population genetic diversity.

In recent years, the rapid advancement of high-throughput sequencing technologies, particularly third-generation sequencing (TGS), has provided a key technical foundation for molecular biology research in areas such as evolutionary analysis, functional gene mining and genomic breeding (Huang et al., 2025). A prime example is Pacific Biosciences’ single-molecule real-time (SMRT) sequencing, which leverages its circular consensus sequencing (CCS) mode to generate long reads and high-fidelity (HiFi) reads (Wenger et al., 2019). These capabilities have substantially enhanced the continuity and completeness of genome assemblies. Meanwhile, high-throughput chromosome conformation capture (Hi-C) technology enables genome-wide DNA interactions to be deciphered, facilitating the construction of high-resolution, three-dimensional chromatin architecture (Dekker et al., 2002). Integrating third-generation long-read sequences with chromatin interaction data enabled us to overcome key challenges associated with repetitive sequences and structural variations in genome assembly (Wenger et al., 2019). Therefore, this study employed a combined strategy of PacBio HiFi sequencing and Hi-C technology. The result was the decoding of a high-quality, chromosome-level genome for *T*. *heterochaetus*. This genome assembly provides the first systematic insights into the gene structure and distribution of functional elements in *T. heterochaetus*, as well as the chromosome arrangement. It also provides foundational data to support subsequent genetic breeding target screening, analysis of adaptive evolution mechanisms, and germplasm resource conservation. Furthermore, this work establishes critical genomic resources for population genetics studies and provides a fundamental molecular basis for evolutionary biology research on Nereididae polychaetes.

## 2 Materials and methods

### 2.1 Sample collection and DNA extraction

**FIGURE 1:**
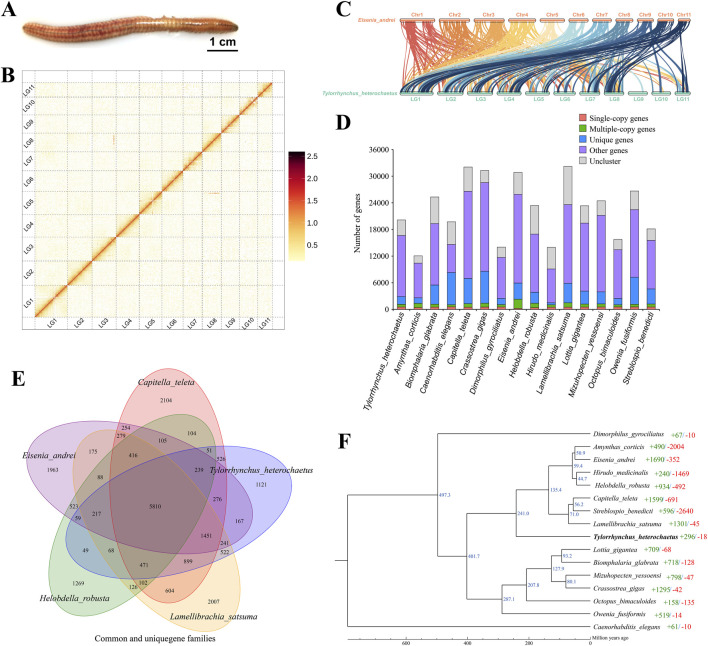
Characteristics of the *Tylorrhynchus heterochaetus* genome. **(A)** The *Tylorrhynchus heterochaetus*. **(B)** Genome-wide Hi-C interaction heatmap of *Tylorrhynchus heterochaetus*. The heatmap illustrates interaction frequencies across the genome, with darker red intensities indicating higher interaction frequencies. The 11 distinct square blocks along the diagonal represent the 11 reconstructed pseudo-chromosomes (linkage groups). The schematic on the right serves as the color scale key for interaction frequency. LG1-11 are the abbreviations of Lachesis Group 1–11, representing the 11 pseudo-chromosomes. **(C)** Genome comparison between *Tylorrhynchus heterochaetus* and *Eisenia andrei*. The lines between the horizontal lines link the alignment blocks. **(D)** Gene family clustering in *Tylorrhynchus heterochaetus* and fifteen other species. **(E)** Venn Diagram representation of gene family overlaps and specificities among *Tylorrhynchus heterochaetus*, *Capitella teleta*, *Eisenia andrei*, *Helobdella robusta* and *Lamellibrachia satsuma*. **(F)** Phylogenetic analysis and a divergence time tree for *Tylorrhynchus heterochaetus* relative to other species were inferred, using *Caenorhabditis elegans as* the outgroup. The number of significantly expanded (+, green) and contracted (−, red) gene families is assigned to each branch following the species names. Estimated species divergence times (in million years ago, Ma) are indicated on each branch; these times were retrieved from the TimeTree database.

One adult male individual of the species *Tylorrhynchus heterochaetus* (body length: 7.5 cm) was obtained from Guangdong Yanghai Agricultural Technology Co., Ltd. (Yangjiang, Guangdong, China; 111°55′23″E, 21°49′16″N; Figure 1A). Following three washes with sterile water, the body wall muscle tissue was dissected from the individual and transferred to a 2 mL cryotube. The tissue was rapidly frozen in liquid nitrogen and stored at −80 °C until required. For Illumina DNA library, fresh muscle tissue was used for DNA extraction using the phenol–chloroform method (Sambrook and Russell, 2006). For PacBio HiFi long-read sequencing, the high-molecular-weight (HMW) DNA was extracted using the Genomic-tip kit (Qiagen, Hilden, Germany) following the manufacturer’s instructions. The DNA concentration and purity were assessed using a Qubit 3.0 Fluorometer (Thermo Fisher Scientific, Waltham, MA, United States) and a NanoDrop spectrophotometer (Thermo Fisher Scientific, Waltham, MA, United States), respectively; the integrity was evaluated using 1.0% agarose gel electrophoresis. All animal procedures were performed in accordance with approval from the Laboratory Animal Ethics Committee of Yangjiang Polytechnic (licence no. 2019DW003).

### 2.2 Library construction and genome sequencing

To characterise the genomic features of *Tylorrhynchus heterochaetus*, an Illumina DNA library with a 350 bp insert size was constructed using the Illumina Genomic DNA Sample Preparation Kit (Illumina, San Diego, CA, United States), following the manufacturer’s protocol. Sequencing was then performed on an Illumina HiSeq X Ten platform by Novogene (Beijing, China) using a 150 bp paired-end protocol.

A DNA library was constructed using the SMRTbell Express Template Prep Kit 2.0 (Pacific Biosciences, Menlo Park, CA, United States) to perform PacBio HiFi long-read sequencing, according to the standard PacBio protocol. The library was then sequenced on a PacBio Sequel II platform. High-accuracy consensus reads were generated using the Circular Consensus Sequencing (CCS) module in SMRT Link v11.0 (Chin et al., 2013).

Approximately 1 g of muscle tissue was harvested for Hi-C sequencing. A Hi-C library was constructed using the GrandOmics Hi-C Kit (GrandOmics, Wuhan, China) in accordance with the manufacturer’s protocol. Briefly, chromatin was crosslinked *in situ* with formaldehyde to preserve 3D chromatin structures, and the reaction was quenched with glycine. Nuclei were subsequently extracted via tissue lysis, and the fixed chromatin was digested with the restriction enzyme *MboI*. Following digestion, DNA ends were filled with biotinylated nucleotides and ligated to generate chimeric molecules. After reversing crosslinks, the DNA was purified and sheared to prepare the sequencing library. Library concentration and insert size were assessed using a Qubit 3.0 Fluorometer (Thermo Fisher Scientific, Waltham, MA, United States) and an Agilent 2,100 Bioanalyzer (Agilent Technologies, Santa Clara, CA, United States), respectively. Sequencing was performed on an Illumina NovaSeq 6,000 platform in paired-end mode. Raw reads were preprocessed using Fastp v0.20.0 (Chen et al., 2018) to remove adapters and filter out low-quality sequences.

### 2.3 Transcriptome sequencing

As muscle tissue constitutes the majority of the body mass of *Tylorrhynchus heterochaetus*, it was selected for transcriptome sequencing. Total RNA was extracted from the tissue using TRIzol reagent (Invitrogen, Carlsbad, CA, United States), following the manufacturer’s protocol. mRNA was then purified from the total RNA extract using an Oligotex mRNA Midi Kit (Qiagen GmbH, Hilden, Germany). The integrity of the RNA was evaluated using an Agilent 2,100 Bioanalyzer, and only samples with an RNA Integrity Number (RIN) of at least 8.0 were retained for library construction. The library was constructed in accordance with the manufacturer’s guidelines and was then sequenced using a 150 bp paired-end protocol on a HiSeq X Ten platform (Illumina, San Diego, California, United States).

### 2.4 Genome size assessment and preliminary assembly

To estimate the genome size, heterozygosity, and repeat content of *Tylorrhynchus heterochaetus*, we performed K-mer analysis using Jellyfish v2.3.0 (Marçais and Kingsford, 2011). The frequency distribution was computed based on 17-mers from clean Illumina sequencing reads. A k-mer size of 17 was selected as this yielded the most consistent and biologically plausible estimates compared to k = 21 or 31. It also offered the clearest distinction between heterozygous and homozygous peaks. The k-mer distribution can be used to estimate genome size, and the peak of the k-mer frequency curve can be used as an indicator of overall sequencing depth. The genome size was calculated using the following formula: Genome size = total k-mer number/peak depth (Marçais and Kingsford, 2011).

In a heterozygous genome, single nucleotide polymorphism (SNP) sites are usually few and far between. Ideally, two heterozygous K-mers are generated around each SNP site. These heterozygous K-mers exhibit half the expected coverage depth of homozygous K-mers. The heterozygosity rate is estimated using the following equation:

$$\text{Heterozygosity rate}=\left(a_{1/2}\times n_{\text{Kspecies}}/\left(2\times K\right)\right)/\left(n_{\text{Kspecies}}-a_{1/2}\times n_{\text{Kspecies}}/2\right)=a_{1/2}\;/\left(K\left(2-a_{1/2}\right)\right).$$

where n_Kspecies_ denotes the total number of K-mer species, and a_1/2_ represents the proportion of heterozygous K-mer species (Li and Waterman, 2003; Liu et al., 2013). Deviations between the observed K-mer distribution and a theoretical Poisson distribution can arise from sequencing errors or copy number variations, both of which can affect subsequent estimates. Therefore, the repeat rate was calculated based on the percentage of total K-mers with depths exceeding 1.8 times the main peak depth (Liu et al., 2013). Following characterisation, the preliminary genome was assembled using PacBio HiFi long-read with hifiasm v0.11 (Cheng et al., 2021) and the default parameters. The resulting contig-level assembly was subsequently polished with Pilon v1.23 (Walker et al., 2014) and clean Illumina short reads to correct potential base-calling errors.

### 2.5 Chromosome-level genome assembly and assessment

To construct a chromosome-level genome assembly, raw Hi-C data were processed using Hicup v0.8.1 (Wingett et al., 2015), which identified valid interaction pairs. These valid pairs were then utilized by the 3D-DNA v180419 pipeline (Dudchenko et al., 2017) to anchor, order, and orient the contigs into pseudo-chromosomes. Visual inspection and manual curation were subsequently performed using Juicebox v1.9.8 (Durand et al., 2016) to correct assembly errors. Following this refinement, the final chromosome-level assembly was generated. To validate scaffold ordering into pseudo-chromosomes, we performed synteny analysis between the genome of *Tylorrhynchus heterochaetus* and the chromosome-level assembly of *Eisenia andrei* using MCScanX (Wang et al., 2012).

To assess the completeness of the assembly, we employed both BUSCO v5.2.2 (Simão et al., 2015) and CEGMA v2.5 (Parra et al., 2007). BUSCO analysis was conducted against the annelida_odb10 lineage dataset, while CEGMA evaluated the coverage of core conserved eukaryotic genes.

### 2.6 Repetitive sequence and noncoding RNA annotation

We employed a hybrid approach combining *de novo* and homology-based methods to identify repeat sequences in the *Tylorrhynchus heterochaetus* genome. For the latter, RepeatMasker v4.1.0 (Bao et al., 2015) was used to identify known repetitive elements. *De novo* repetitive element databases were constructed using LTRharvest v1.0.6 (Zhao and Wang, 2007), RepeatScout v1.0.5 (Abrusán et al., 2009) and RepeatModeler v2.0.1 (Price et al., 2005). Tandem repeats were also predicted using TRF v4.09 (Benson, 1999). Non-coding RNAs (ncRNAs) were annotated using tRNAscan-SE v2.0 (Chan et al., 2021) and Infernal v1.1.3 (Nawrocki and Eddy, 2013), which identified tRNAs, rRNAs, snRNAs and miRNAs.

### 2.7 Gene prediction and functional annotation

We employed a comprehensive strategy integrating homology-based, transcript-based and *de novo* prediction methods to predict protein-coding genes. For *de novo* prediction, we utilised five tools: AUGUSTUS v3.2.3 (Stanke and Waack, 2003); GlimmerHMM v3.04 (Majoros et al., 2004); SNAP v2013-11-29 (Korf, 2004); GeneID v1.4 (Blanco et al., 2007); and Genscan v3.1 (Burge and Karlin, 1997). These were used to identify candidate genes. Homology-based predictions were conducted using protein sequences retrieved from the publicly available databases for the following six species: *Helobdella robusta* (GCF_000326865.1), *Lamellibrachia satsuma* (GCA_022478865.1), *Dimorphilus gyrociliatus* (GCA_904063045.1), *Eisenia andrei* (GWHACBE00000000), *Capitella teleta* (GCA_000328365.1) and *Owenia fusiformis* (GCA_903813345.2). Homology searches and gene annotation were then performed using GeMoMa v1.6.4 (Keilwagen et al., 2016) with the default parameters. For transcript-based prediction, short-read Illumina RNA-seq data were assembled into transcripts using Trinity v2.5.2 (Haas et al., 2013) and the resulting gene structures were validated using PASA v2.3.3 (Haas et al., 2003). Finally, all gene predictions were integrated via the Evidence Modeler (EVM) pipeline v1.0 (Haas et al., 2008) to produce a consensus gene set.

Gene functional annotation involved aligning protein sequences to the Swiss-Prot database (Bairoch and Apweiler, 2000) using BLASTp (Altschul et al., 1990), and functional assignments were derived from the best hit (e-value ≤1 × 10^−5^). Motif and domain annotations were performed using InterProScan v5.31 (Quevillon et al., 2005), which queries public databases including ProDom, PRINTS, Pfam, SMART, PANTHER and PROSITE. Gene Ontology (GO) terms (Ashburner et al., 2000) were then assigned to each gene by mapping them to the relevant InterPro entries (Finn et al., 2017). Protein-coding genes were further annotated by transferring functional labels from two sources: (1) the top BLASTp hits (e-value <1 × 10^−5^) in the Swiss-Prot database, and (2) the DIAMOND hits (e-value <1 × 10^−5^) in the non-redundant protein database (Kanz et al., 2005; Buchfink et al., 2015). Additionally, the gene set was mapped to Kyoto Encyclopedia of Genes and Genomes (KEGG) pathways (Kanehisa and Goto, 2000) to assign each gene to the most appropriate metabolic or signalling pathway.

### 2.8 Gene family identification and phylogenetic analysis

We used OrthoMCL v2.0.9 (Li et al., 2003) to identify orthologous groups based on protein sequences from sixteen species: *Dimorphilus gyrociliatus* (Annelida, Dinophilidae, GCA_904063045.1), *Amynthas corticis* (Annelida, Megascolecidae, GWHAOSM00000000.1), *Eisenia andrei* (Annelida, Lumbricidae, GWHACBE00000000), *Hirudo medicinalis* (Annelida, Hirudinidae, GCA_011800805.1), *Helobdella robusta* (Annelida, Glossiphoniidae, GCF_000326865.1), *Capitella teleta* (Annelida, Capitellidae, GCA_000328365.1), *Streblospio benedicti* (Annelida, Spionidae, GCA_019095985.1), *Lamellibrachia satsuma* (Annelida, Siboglinidae, GCA_022478865.1), *Owenia fusiformis* (Annelida, Oweniidae, GCA_903813345.2), *Tylorrhynchus heterochaetus* (Annelida, Nereididae, GWHHJEW00000000.1), *Lottia gigantea* (Mollusca, Lottiidae, GCF_000327385.1), *Biomphalaria glabrata* (Mollusca, Planorbidae, GCF_947242115.1), *Mizuhopecten yessoensis* (Mollusca, Pectinidae, GCF_002113885.1), *Crassostrea gigas* (Mollusca, Ostreidae, GCF_000297895.1), *Octopus bimaculoides* (Mollusca, Octopodidae, GCA_001194135.2) and *Caenorhabditis elegans* (Nematoda, Rhabditidae, GCF_000002985.6). These species were selected based on their phylogenetic representation within Annelida and Lophotrochozoa, as well as the availability of high-quality reference genomes. Selected species included key annelid references, such as *Eisenia andrei*, which serve as critical benchmarks for understanding annelid evolution. The genes were then grouped into orthologous (orthologs) and paralogous (paralogs) clusters. Single-copy orthologues shared by all 16 species were aligned using MUSCLE v3.8.31 (Edgar, 2004) and a maximum likelihood (ML) phylogenetic tree was reconstructed using PHYML v3.0 (Guindon et al., 2010). Divergence times among species were estimated using the MCMCTree program (PAML package v4.7a; Yang, 2007), with calibration based on divergence time estimates from the TimeTree database (http://timetree.org/).

### 2.9 Gene family comparison

Gene family expansion and contraction are key drivers of phenotypic diversity and environmental adaptation (Rayna and Hans, 2015). In this study, we used CAFE v4.2 (De Bie et al., 2006) to identify gene families that had expanded or contracted significantly (adjusted *p* < 0.05). We estimated ancestral gene family numbers using a birth-death model to infer evolutionary expansions and contractions. To elucidate the biological functions associated with these changes, we performed KEGG pathway enrichment analysis using Fisher’s exact test, applying a False Discovery Rate (FDR) correction (<0.05) to control for multiple testing.

To identify putative positively selected genes (PSGs) and genes undergoing accelerated evolution, we constructed a set of single-copy orthologues common to five species: *Capitella teleta*, *Eisenia andrei*, *Helobdella robusta*, *Owenia fusiformis* and *Lamellibrachia satsuma*. First, we conducted multiple sequence alignments of protein sequences within each gene family via Muscle v3.6 (Edgar, 2004). Synonymous (Ks) and non-synonymous (Ka) substitution rates were then calculated using PAML v4.4c (Yang, 1997), with codon-based substitution models implemented via the codeml program and the likelihood ratio test (LRT). To further pinpoint PSGs, we applied the branch-site model, computing LRT *p*-values under the null hypothesis of a 50:50 mixture between an ω^2^-distribution (df = 1) and a point mass at zero. Stringent filtering was subsequently applied, retaining only genes that satisfied all of the following criteria: (a) gene length ≥300 bp, (b) ≥2 positively selected sites, and (c) no gaps in any stretch of three consecutive amino acids.

## 3 Data

### 3.1 Genome sequencing and assembly

K-mer analysis (K = 17) based on Illumina reads indicated a sequencing depth of ∼57×, estimating the *Tylorrhynchus heterochaetus* genome size at 759.53 Mb, with a heterozygosity rate of 1.41% and a repeat content of 45.92% (Supplementary Figure S1). PacBio HiFi sequencing generated 20 Gb of HiFi reads (Supplementary Table S1). As an annelid, *T. heterochaetus* possesses tissues rich in polysaccharides and mucus, which are prone to co-precipitation during HMW DNA extraction. Consequently, although the yield of ultra-HMW DNA was limited, the quantity obtained met the requirements for library construction and assembly. Subsequently, we generated a primary contig assembly of 782.25 Mb with a contig N50 of 29.29 Mb using hifiasm (Table 1). Finally, by integrating Hi-C data, a total of 777.18 Mb scaffolds (representing 99.35% of the total sequence) was anchored onto 11 linkage groups (Supplementary Table S2; Figure 1B), yielding a highly continuous final assembly with a scaffold N50 of 75.39 Mb. Furthermore, synteny analysis revealed a clear correspondence between the 11 linkage groups of *T. heterochaetus* and the chromosomes of *Eisenia andrei*, thereby validating the ordering of scaffolds into pseudo-chromosomes (Figure 1C).

**TABLE 1:** Statistics of *Tylorrhynchus heterochaetus* genome assembly and annotation data.

Category	Statistics
Assembly	
Scaffold assembly size (bp)	782,252,994
Number of scaffolds	58
Scaffold N50 (bp)	75,387,155
Longest scaffold (bp)	104,054,433
Contig assembly size (bp)	782,246,894
Number of contigs	119
Contig N50 (bp)	29,288,192
Longest contig max (bp)	98,377,711
Genome BUSCO (% of total BUSCO)	
Complete	1,677 (96.5%)
Single-copy	1,649 (94.9%)
Duplicated	28 (1.6%)
Fragmented	17 (1.0%)
Missing	44 (2.5%)
CEGMA	
CEGs (% of all CEGs)	239 (96.37%)
Repetitive sequences (% of genome)	
SINE (bp)	185,451 (0.02%)
LINE (bp)	26,061,701 (3.33%)
LTR (bp)	368,972,832 (47.17%)
DNA (bp)	11,398,584 (1.46%)
Unclassified (bp)	16,333,421 (2.09%)
Total (bp)	398,320,436 (50.92%)
Gene annotations (% of all genes)	
Nr annotation	18,503 (91.8%)
KEGG annotation	15,389 (76.4%)
InterPro annotation	18,203 (90.4%)
GO annotation	11,203 (55.6%)
Pfam annotation	13,930 (69.1%)
All annotated	19,395 (96.3%)
Total gene number	20,145 (100%)
Gene BUSCO (% of total BUSCO)	
Complete	1,670 (96.1%)
Single-copy	1,628 (93.7%)
Duplicated	42 (2.4%)
Fragmented	33 (1.9%)
Missing	35 (2.0%)
Non-coding protein genes (% of genome)	
Number of miRNA	2,312
Number of tRNA	1,926
Number of rRNA	3,392
Number of snRNA	262
Length of miRNA	234,532 (0.029982%)
Length of tRNA	152,378 (0.019479%)
Length of rRNA	338,288 (0.043245%)
Length of snRNA	41,163 (0.005262%)

BUSCO assessment revealed 1,677 (96.5%) complete BUSCOs, comprising 1,649 single-copy and 28 duplicated entries (Table 1). The CEGMA analysis identified 239 conserved eukaryotic core genes, constituting 96.37% of the 248 genes in the database (Table 1). These results demonstrate that the genome assembly achieves high coverage and completeness.

### 3.2 Genome annotation

*De novo* prediction and Repbase database analyses revealed that repetitive sequences constitute 54.73% of the *Tylorrhynchus heterochaetus* genome. Among these transposable elements, long terminal repeats (LTRs, 47.17%) were the most abundant, followed by long interspersed nuclear elements (LINEs, 3.33%), DNA transposons (1.46%), and short interspersed nuclear elements (SINEs, 0.02%) (Table 1; Supplementary Table S3).

A combined approach using *de novo*, homology-based, and RNA-seq predictions identified 20,145 protein-coding genes in the *T. heterochaetus* genome, with an average gene length of 10,909.40 bp (Supplementary Table S4). The length distributions of genes, coding sequences (CDS), exons, and introns in *T. heterochaetus* were comparable to those of other annelids (Supplementary Figure S2). The functions of the protein-coding genes were annotated in NR, KEGG, InterPro, GO and Pfam databases. A total of 19,395 genes were annotated, accounting for 96.30% of all protein-coding genes (Table 1).

Noncoding RNAs were predicted using Rfam, cmsearch, and tRNAscan-SE, yielding 2,312 miRNAs, 1,926 tRNAs, 3,392 rRNAs, and 262 snRNAs (Table 1; Supplementary Table S5).

### 3.3 Comparative genome analysis

A comparison of 15 species genomes was conducted to investigate the phylogenetic relationships of *Tylorrhynchus heterochaetus*. These species were selected based on their phylogenetic representation within Annelida and Lophotrochozoa, as well as the availability of high-quality reference genomes. They include close relatives such as *Eisenia andrei,* which serve as key references for understanding annelid evolution. In total, 33,522 gene families were clustered across the 16 species, with *T. heterochaetus* possessing 12,170 of these gene families (Figure 1D; Supplementary Table S6). A venn diagram was constructed for the gene families of *Capitella teleta*, *Eisenia andrei*, *Helobdella robusta, Lamellibrachia satsuma* and *T. heterochaetus*. Among these, 1,121 gene families were unique to *T. heterochaetus*, while 5,810 gene families were conserved across all five species (Figure 1E).

The ML phylogenetic tree was constructed from single-copy orthologs. The *Caenorhabditis elegans* as an outgroup in a separate clade, while the remaining 15 Lophotrochozoa species form a single clade (Figure 1F). This Lophotrochozoa clade further resolves into three distinct branches. The *Dimorphilus gyrociliatus* diverged from the common ancestor of the other 14 species at approximately 497.3 Ma. Subsequently, around 401.7 Ma, the common ancestor of eight Annelida species (*Amynthas corticis*, *E*. *andrei*, *Hirudo medicinalis*, *H*. *robusta*, *C*. *teleta*, *Streblospio benedicti*, *L*. *satsuma*, and *T. heterochaetus*) separated from the ancestor of the remaining six species (*Lottia gigantea*, *Biomphalaria glabrata*, *Mizuhopecten yessoensis*, *Crassostrea gigas*, *Octopus bimaculoides*, *Owenia fusiformis*). Notably, the annelid *O*. *fusiformis* clusters with the five Mollusca species. Recent studies indicate that Oweniidae and Magelonidae form a monophyletic group, termed Palaeoannelida, which constitutes the sister taxon to all other annelids (Weigert and Bleidorn, 2016), potentially explaining this unusual placement. In our analysis, *T. heterochaetus*, an annelida from Polychaeta, Errantia, clusters with Oligochaeta (*A*. *corticis* and *E*. *andrei*), Hirudinea (*H*. *medicinalis* and *H*. *robusta*), and Sedentaria (*C*. *teleta*, *S*. *benedicti*, and *L*. *satsuma*) species, all of which belong to Annelida. This consistent taxonomic grouping underscores the high accuracy of the genome assembly presented here.

Gene family expansion and contraction represent key drivers of phenotypic diversity evolution. We compared gene families across sixteen species: *C. elegans*, *D. gyrociliatus*, *A. corticis*, *E. andrei*, *H. medicinalis*, *H. robusta*, *C. teleta*, *S. benedicti*, *L. satsuma*, *T. heterochaetus*, *L. gigantea*, *B. glabrata*, *M. yessoensis*, *C. gigas*, *O. bimaculoides*, and *O. fusiformis*. In *T. heterochaetus*, 296 gene families were significantly expanded (*p* < 0.05) and 18 were significantly contracted (*p* < 0.05) (Supplementary Table S7). KEGG enrichment analysis demonstrated that expanded gene families were preferentially associated with signal transduction, immune response, and digestive processes. Key enriched pathways included cAMP and TNF signaling, cytosolic DNA-sensing, ABC transporters, and vitamin digestion. Notably, the expansion of the GPCR, TLR, and Cytochrome P450 superfamilies likely facilitates adaptation to the challenges of the benthic environment (Supplementary Table S8). In contrast, contracted lineages were largely restricted to functional categories involving cellular community (e.g., gap/tight junctions) and developmental processes (e.g., dorso-ventral axis formation and axon regeneration) (Supplementary Table S9). Additionally, positive selection occurs when non-synonymous amino acid mutations arise in single-copy gene families as organisms adapt to external influences. Using *C. teleta*, *E. andrei*, *H. robusta*, *O. fusiformis*, and *L. satsuma* as controls, we identified genes under positive selection in *T. heterochaetus*. The analysis revealed 305 genes under positive selection (Supplementary Table S10).

## 4 Conclusion

This study presents a chromosomal-level genome assembly for *Tylorrhynchus heterochaetus*. The resulting genome exhibits continuity and completeness comparable to other high-quality Annelida genomes, thereby offering a valuable reference for systems biology and comparative evolutionary analysis. This reference genome holds importance for the aquaculture and artificial breeding of *T. heterochaetus*, establishing a foundation for further investigation.

## Data Availability

The original contributions presented in the study are publicly available. The final genome assembly and annotation files (predicted CDS and protein sequences) generated in this study have been deposited in the Figshare repository (https://doi.org/10.6084/m9.figshare.31015993). Raw sequencing data, including Illumina, PacBio, Hi-C, and RNA-seq reads, have been deposited in the NCBI Sequence Read Archive (SRA) under BioProject PRJNA1346694 (Accession numbers: SRR35822427, SRR35819296, SRR35859756, and SRR35859347). Additionally, the final genome assembly is available in the Genome Warehouse (GWH) of the National Genomics Data Center (NGDC) under accession number GWHHJEW00000000.1 (BioProject PRJCA049339).
