# Supplementary figures and images for: Chromosome-level reference genome of Tylorrhynchus heterochaetus (Annelida, Nereididae)
Source: Front Genet. 2026 Jan 28;17:1753621. doi: 10.3389/fgene.2026.1753621 (PMC12890243; doi:10.3389/fgene.2026.1753621)

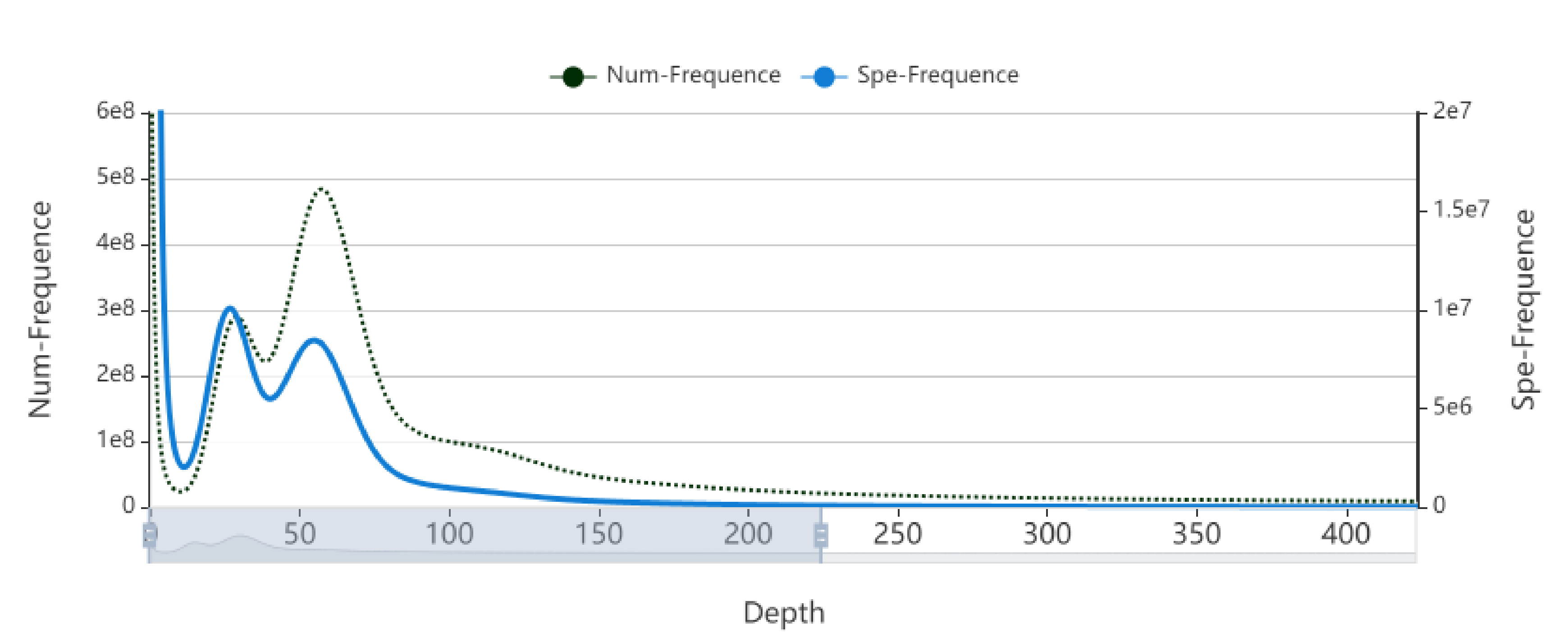

Supplement: Supplementary file 1 [file Image1.jpeg]

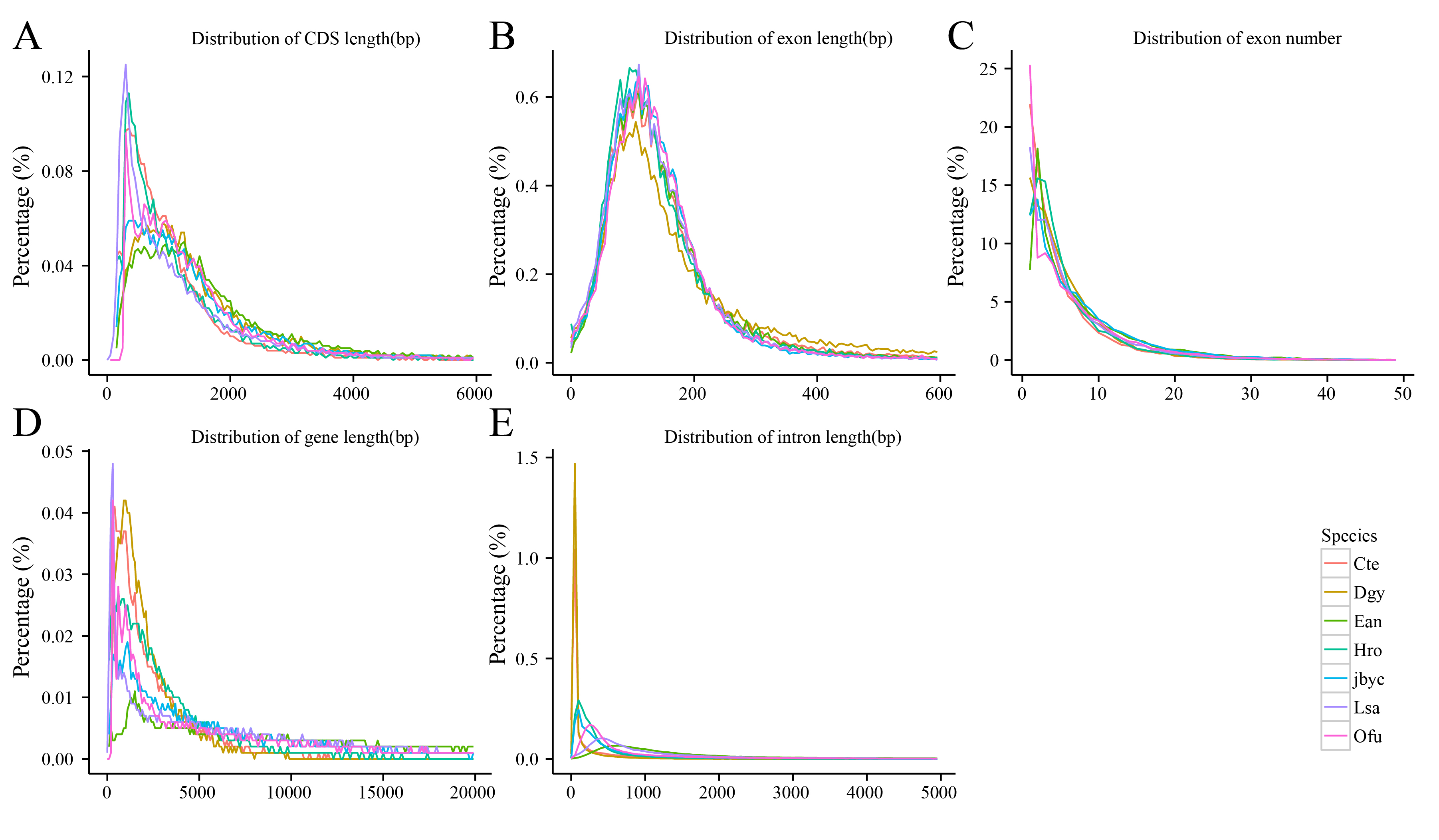

Supplement: Supplementary file 2 [file Image2.jpeg]
